# Alternative Splicing in Adhesion- and Motility-Related Genes in Breast Cancer

**DOI:** 10.3390/ijms17010121

**Published:** 2016-01-16

**Authors:** Rosanna Aversa, Anna Sorrentino, Roberta Esposito, Maria Rosaria Ambrosio, Angela Amato, Alberto Zambelli, Alfredo Ciccodicola, Luciana D’Apice, Valerio Costa

**Affiliations:** 1 Institute of Genetics and Biophysics “Adriano Buzzati-Traverso”, Consiglio Nazionale delle Ricerche, Via P. Castellino 111, 80131 Naples, Italy; rosanna.aversa@gmail.com (R.A.); anna.sorrentino@igb.cnr.it (A.S.); roberta.esposito@igb.cnr.it (R.E.); mariarosaria.ambrosio@gmail.com (M.R.A.); alfredo.ciccodicola@igb.cnr.it (A.C.); 2 Lab of Experimental Oncology and Pharmacogenomics, Istituto di Ricovero e Cura a Carattere Scientifico Fondazione “Salvatore Maugeri”, 27100 Pavia, Italy; angela.amato@fsm.it; 3 Medical Oncology Unit, Azienda Ospedaliera Papa Giovanni XXIII, 24127 Bergamo, Italy; alberto.zambelli@fsm.it; 4 Department of Science and Technology, University Parthenope of Naples, 80131 Naples, Italy; 5 Institute of Protein Biochemistry, Consiglio Nazionale delle Ricerche, Via P. Castellino 111, 80131 Naples, Italy; l.dapice@ibp.cnr.it

**Keywords:** alternative splicing, breast cancer, RNA-Sequencing, cell adhesion and motility

## Abstract

Breast cancer is the most common tumor and the second leading cause of cancer death among woman, mainly caused by the metastatic spread. Tumor invasiveness is due to an altered expression of adhesion molecules. Among them, semaphorins are of peculiar interest. Cancer cells can manipulate alternative splicing patterns to modulate the expression of adhesion- and motility-related molecules, also at the isoform level. In this study, combining RNA-Sequencing on MCF-7 to targeted experimental validations—in human breast cell lines and breast tumor biopsies—we identified 12 new alternative splicing transcripts in genes encoding adhesion- and motility-related molecules, including semaphorins, their receptors and co-receptors. Among them, a new *SEMA3F* transcript is expressed in all breast cell lines and breast cancer biopsies, and is translated into a new semaphorin 3F isoform. *In silico* analysis predicted that most of the new putative proteins lack functional domains, potentially missing some functions and acquiring new ones. Our findings better describe the extent of alternative splicing in breast cancer and highlight the need to further investigate adhesion- and motility-related molecules to gain insights into breast cancer progression.

## 1. Introduction

Breast cancer (BC) is the most common tumor among women, and the second leading cause of cancer death [1]. Its progression is accompanied by genomic alterations including inherited genetic variations, acquired (*i.e.*, *de novo*) genomic aberrations, changes in gene expression, splicing pattern and—in some cases—in protein functionality. Cancer cells can modulate, even through alternative splicing (AS), the expression and the variety of adhesion/motility genes, accumulating transcripts with potentially different or opposite functions [2]. As in most of cancers, the major cause of death in BC patients is due to metastatic spread of cells from the primary tumor [3], through the destabilization of intercellular contacts and the acquisition of mobilization- and invasion-related features [4]. Proteins involved in cell-cell/cell-matrix adhesion and cell motility and migration—including the emerging class of semaphorins [5]—are crucial players in the metastatic process. Thus, they are optimal biomarkers of cancer progression and prognostic indicators, as well as potential candidates to design therapeutic strategies. Since isoform switching in adhesion and motility proteins is one of cancer hallmarks [6], investigating the AS pattern in non-metastatic tumor cells (*i.e.*, before epithelial-to-mesenchymal transition, EMT) may help to address how they develop a more aggressive and invasive phenotype. RNA-Sequencing (RNA-Seq) is the most powerful approach to investigate the transcriptome of cancer cells allowing to explore AS, mutations and RNA editing [7,8]. In light of this, we performed a systematic transcriptome analysis in the MCF-7 cell line, a widely used cellular model of human luminal breast tumors responsive to endogenous hormones. In particular, using RNA-Seq, we simultaneously investigated the expression and the AS pattern of genes that encode adhesion and motility-related molecules, including semaphorins, their receptors and co-receptors. Combining a previously developed computational approach for new AS events [9] to targeted experimental validations, we identified 12 new transcripts of genes encoding adhesion- and motility-related proteins. Most of them are predicted to translate proteins with different functions compared to the canonical isoforms. Interestingly, they are expressed in BC cell lines and—at a lower and variable extent—in BC biopsies. Among them, a new shorter SEMA3F alternative isoform, highly expressed in BC cell lines and breast cancer biopsies, was identified.

## 2. Results

### 2.1. MCF-7 Transcriptome and Alternative Splicing Profile

**Figure 1:**
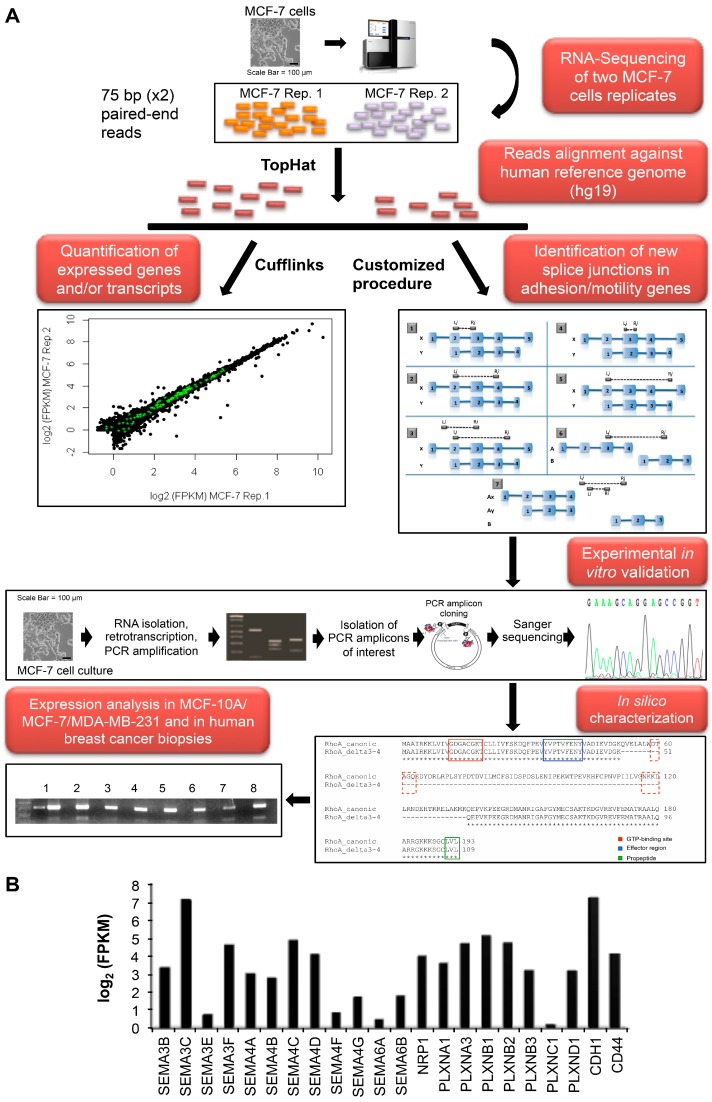
(**A**) Overview of RNA-Seq data processing and of the computational and experimental approach used for the identification and validation of the new spliced transcripts; (**B**) Barplot with log_2_ normalized expression values (FPKM) of semaphorin/Plexin/Neuropilin—and of the adhesion markers *CDH1* and *CD44*—according to RNA-Seq data.

To comprehensively study the expression—and the alternative splicing pattern—of genes encoding adhesion- and motility-related proteins in breast cancer, we used RNA-Seq to profile the transcriptome of MCF-7 cells as a model of human luminal breast tumor responsive to estrogen stimulation. A schematic workflow of our computational and experimental procedures is depicted in Figure 1A. As confirmed by RNA-Seq, these cells have a low metastatic ability and did not undergo EMT, representing a good model to study earlier stages of BC progression. Indeed, expression data indicated high levels of *CDH1* (E-cadherin) and almost undetectable levels of *CDH2* and *VIM* that encode mesenchymal markers N-cadherin and vimentin, respectively (Figure 1B). The intermediate expression levels of *CD44* and the absence of *CD24* are in agreement with [10], which demonstrated that the low colony forming activity of MCF-7 is accompanied by increased adhesive properties of these cells, due to the described expression pattern (Figure 1B).

As we were interested in a systematic analysis of adhesion/motility-related genes, the GeneCards database (version 3.12) was used to retrieve a list of genes involved in “cell adhesion”, “cell–matrix interaction” and “cell motility” processes. The analysis of RNA-Seq data (mapping summary in Figure S1) revealed that MCF-7 cells express 1217 adhesion- and motility-related genes (Figure S2) and 20 genes encoding semaphorins and their receptors, plexins and neuropilins (Figure 1B), that are associated with the onset/progression of different cancer types [5,11,12]. Among them, *SEMA3F*, *SEMA4C*, *PLXNB1* and *PLXNB2*, involved in cancer progression and in immune system suppression [11,13], were highly expressed. Using a previously published computational procedure [9], 17 new exon-skipping events in 14 adhesion/motility-related genes were identified (Figure S3). The presence of 12 out of 17 new transcripts identified by RNA-Seq analysis was *in vitro* confirmed by RT-PCR, cloning (where needed) and Sanger sequencing (Figure S2). Details are reported in the Supplementary File and in Figures S4–S14. Selected candidate genes were classified according to their functions in (1) adhesion-; (2) motility-related and (3) semaphorins and their receptors. The results are schematized in Table 1.

To assess if and at what extent these new transcripts were expressed in breast and if they could be expressed also in other breast cell lines and *in vivo*, we systematically measured the mRNA levels in a non-tumorigenic breast cell line (MCF-10A), in MCF-7, in a triple negative aggressive metastatic BC cell line (MDA-MB-231) and, particularly, in BC biopsies.

**Table 1:** Summary of new validated transcripts and of their predicted proteins.

Gene Name	Positions	ID	Skipped Exons	ORF Change	Affected Domain(s)	Protein Length
*THBS1*	15q14	LN607833	10	Del	TSP1	1112
*LGALS1*	22q13.1	LN607840	3	Frameshift	β-galactoside binding	39
*RAP1GAP*	1p36.12	LN607839	24	Ins	*N*/*A*	821
*CD47*	3q13.12	LN680437	8–10	Frameshift	Cytoplasmic C-term	293
*RHOA*	3p21.31	LN607834	3–4	Del	GTP binding	109
*RHOD*	11q13.2	LN607835	4	Del	GTP binding	165
*CASK*	Xp11.4	LN607837	19–20	Del	Linker between PDZ and SH3	886
*CTTN*	11q13.3	LN607836	10–11	Del	Cortactin	476
*JAG2*	14q32.33	LN607838	24	Del	VWFC	1194
*SEMA3C*	7q21.11	LN626689	15	Frameshift	Type 1 Ig-like and R/K rich	514
*SEMA3F*	3p21.31	LN626688	16	Frameshift	Type 1 Ig-like and R/K rich	571
*PLXNB1*	3p21.31	LN626690	32	Frameshift	Cytoplasmic C-term	1876

ORF, Open Reading Frame; TSP1, Thrombospondin type 1; N/A, Not Applicable; GTP, Guanosine TriPhosphate; PDZ, Postsynaptic density 95, Disc large, Zonula occludens-1; SH3, SRC Homology 3; VWFC, Von Willebrand factor type C; R/K, Arginin/Lysine.

### 2.2. New Transcripts of Adhesion-Related Proteins

As above mentioned, using RNA-Seq data we selected 17 potentially new exon-skipping events in 14 adhesion/motility-related genes for the experimental validation. Specifically, through this analysis we selected four new transcripts of the adhesion-related genes *THBS1* (thrombospondin-1), *RAP1GAP* (RAP1 GTPase Activating Protein), *LGALS1* (galectin 1) and *CD47* (Cluster of Differentiation 47). All of them were experimentally confirmed in BC cell lines.

**Figure 2:**
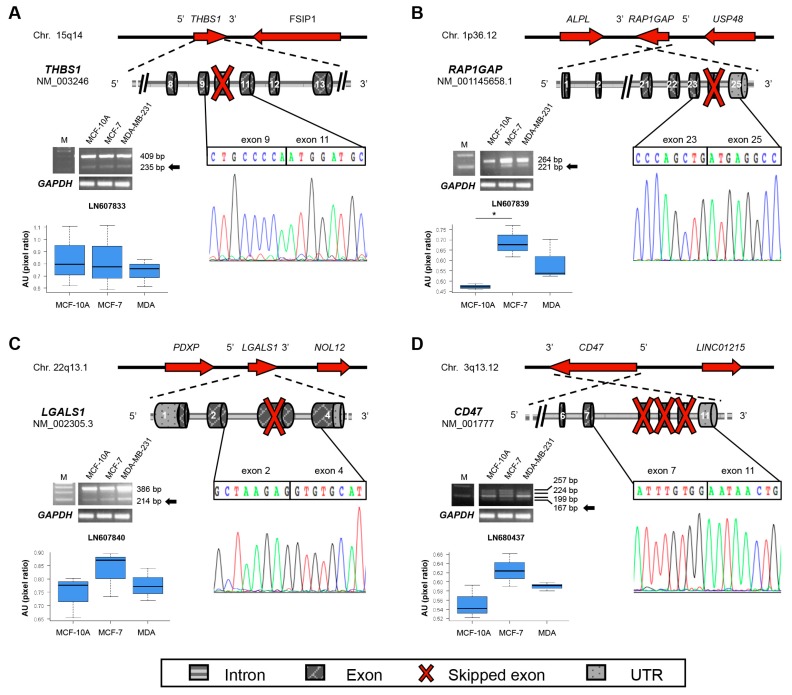
Schematic representation of *de novo* identified transcripts for adhesion-related genes and their differential expression by RT-PCR in breast cell lines (a representative image is shown for each gene). (**A**) *THBS1* new transcript; (**B**) *RAP1GAP* new transcript; (**C**) *LGALS1* new transcript; (**D**) *CD47* new transcript. For each new transcript, in the upper part is shown the genomic region encompassing the gene; red arrows indicate the direction of transcription. Below, the exon/intron structure of the gene is depicted. Red cross indicates the exons that are spliced out in the new transcript. The electropherogram and the nucleotide sequence above refer to the new splice junction identified. Images of PCR amplicons on agarose gel from three independent replicates experiments were acquired. For all panels, M is 100 bp DNA marker. The black arrows indicate the PCR band corresponding to the newly identified transcripts. Pixel density of each band (only for the newly identified mRNA isoforms) was quantified by ImageJ software and compared to the pixel density of the 100 bp DNA marker (at known concentration) to normalize data across replicates and cell lines. GAPDH gel bands are reported below to show that the starting amount of template was the same for all analyzed samples. Values are reported as AU = Arbitrary Units in the boxplots. Dark horizontal lines represent the mean, the box represents the 25th and 75th percentiles and the whiskers the 5th and 95th percentiles. Asterisks indicate significant *p* values (* *p* < 0.05).

In detail, the new transcript (accession no. LN607840) encoding thrombospondin-1 (*THBS1*) lacks the exon 10 (Figure 2A) and it is predicted to encode a protein of 1112 amino acids. Thrombospondin-1 is a 1170 amino acids multi-domain protein that mediates cell-to-cell and cell-to-matrix interactions [14]. It is characterized by a von Willebrand Factor C domain, three TSP (Thrombospondin) type 1 domains, two EGF (Epidermal Growth Factor) like domains, five TSP type 3 domains and a heparin binding region. The newly identified isoform lacks two TSP type 1 domains and few residues of the EGF-like domain (Figure S4). No significant differences in its expression were measured among analyzed breast cell lines (Figure 2A).

Conversely, the new *RAP1GAP* isoform has significantly higher expression in MCF-7 than in normal cells (Figure 2B). Rap1GAP can inhibit cell proliferation and migration acting as a specific negative regulator of Rap1 [15]. The new transcript (accession no. LN607839) is generated by the skipping of exon 24—as predicted by RNA-Seq—which determines the canonical stop codon loss and the formation of a new one in the exon 25. This splicing event is predicted to produce a new protein of 821 residues, with additional 94 residues at the *C*-terminus (Figure S5). These residues are evolutionarily conserved, indicating a potential new function. However, the lack of protein structures in Protein Data Bank (PDB) database hampered any 3D prediction for this new domain.

We also identified a new transcript of galectin-1 (*LGALS1*), generated by the skipping of exon 3. The new transcript (accession no. LN607840) has a higher, though not significant, expression in MCF-7 cell line compared to MCF-10A cell line (Figure 2C). Galectin-1 is an evolutionarily conserved β-galactoside-binding lectin that regulates endothelial cell migration, proliferation, and adhesion [16]. The new transcript has a premature termination codon, and the predicted protein would lack 96 aa, corresponding to two β-galactoside binding regions (Figure S6). It suggests a potential new function for this novel galectin-1 alternative isoform.

Finally, RNA-Seq indicated a new *CD47* transcript (accession no. LN680437) generated by exons 8–10 skipping, expressed at higher but not significant levels in BC cell lines (Figure 2D and Figure S7). Notably, although this transcript is not annotated in public repositories, browsing the literature we found that Reinhold and colleagues [17] identified it in 1995. This isoform was not further analyzed.

### 2.3. New Transcripts of Motility-Related Proteins

**Figure 3:**
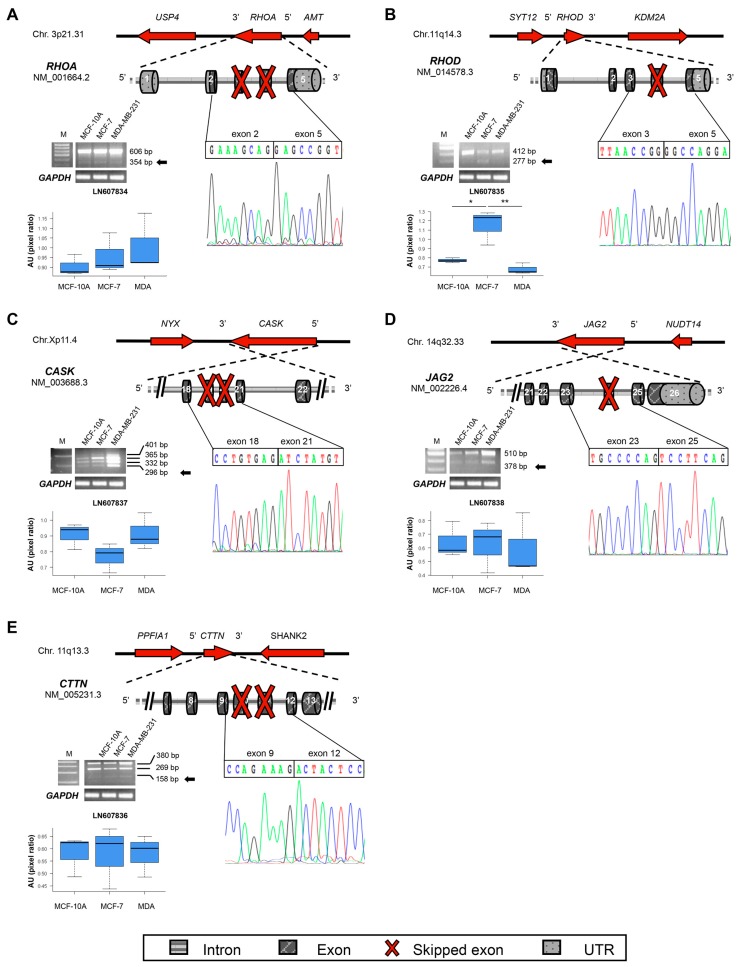
Schematic representation of *de novo* identified transcripts of motility-related genes and their expression by RT-PCR in breast cell lines (a representative image is shown for each gene). (**A**) *RHOA*; (**B**) *RHOD*; (**C**) *CASK*; (**D**) *JAG2*; (**E**) *CTTN* new transcripts. Details about the gel bands and their quantification are provided in Figure 2 legend. Asterisks indicate significant *p* values (* *p* < 0.05; ** *p* < 0.01).

Similarly to adhesion-related molecules, we identified—and experimentally validated—five new motility-related transcripts (Figure 3). They are all generated by exon skipping of modular exons.

In detail, we discovered two novel isoforms of *RHOA* (Ras homolog gene family, member A) and *RHOD* (Ras homolog gene family,member D). RhoA and RhoD are two small (~21 kD) GTPase proteins, characterized by three GTP binding domains. Rho GTPases are key operators in signaling transduction pathways that control cell behavior in response to signals from the extracellular environment [18]. Expression of the new *RHOA* transcript (accession no. LN607834) does not differ among breast cell lines (Figure 3A), while new *RHOD* mRNA (accession no. LN607835) has a significantly higher expression in MCF-7 compared to MCF-10A cell line (Figure 3B). They are predicted to encode novel proteins with potentially new functions. Indeed, if translated, the new *RHOA* transcript would encode a shorter protein of 109 aa that lacks two GTP binding sites (Figure S8). Likewise, the new RhoD isoform would consist of 165 amino acids, lacking a GTP binding site (Figure S9).

In addition, we identified a new transcript (accession no. LN607837) of the calcium/calmodulin-dependent serine protein kinase (*CASK*) gene generated by exon skipping of the exons 19 and 20 (Figure 3C). This new transcript has the highest expression in the most aggressive BC cells (Figure 3C). The calcium/calmodulin-dependent serine protein kinase belongs to the membrane-associated guanylate kinase protein family, whose members function as multiple domain adaptors originally identified at cell junctions and synapses [19]. The new predicted isoform would consist of 886 residues, lacking 35 aa of a linker region between PDZ and SH3 domain (Figure S10). Its potential translation into a new protein isoform is supported by the presence similar protein isoforms, currently annotated in the UniProt database.

A new transcript (accession no. LN607838) of jagged-2 (*JAG2*) gene, one of the ligands of Notch receptor, lacking exon 24 was also identified (Figure 3D). Although its expression is strongly upregulated at the hypoxic invasive front in breast cancers samples [20], we did not observe significant difference in the expression levels of the new *JAG2* transcript in BC cells *vs.* non-tumoral cell line (Figure 3D). The new predicted Jagged-2 protein misses few residues (984–994) that belong to Von Willebrand Factor C domain (Figure S11). However, the computational analysis did not predict any damaging effect on protein functionality.

Finally, we disclosed a new cortactin (*CTTN*) transcript, whose expression did not differ among analyzed cell lines (Figure 3E and Figure S12). Similarly to *CD47*, it is not annotated in public repositories, although van Rossum [21] described it in cell lines and human tissues. We did not further analyze this transcript.

### 2.4. New Semaphorins and Plexins Isoforms

**Figure 4:**
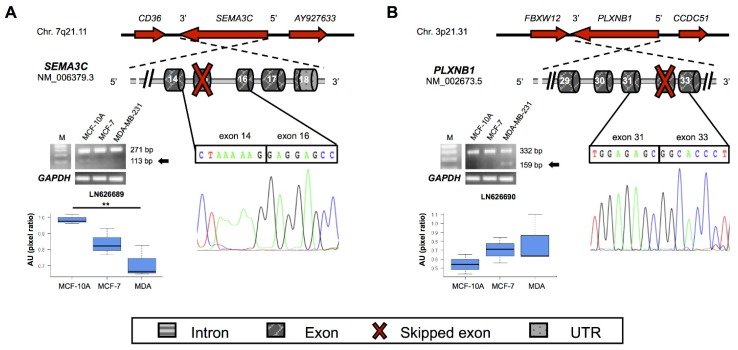
Schematic representation of *de novo* identified transcripts for (**A**) *SEMA3C* (**B**) *PLXNB1* genes. Nucleotide sequenceand their expression by RT-PCR in breast cell lines are also depicted. Details about the gel bands and their quantification are provided in Figure 2’s legend. Asterisks indicate significant *p* values (** *p* < 0.01).

All the exon skipping events identified by RNA-Seq in genes encoding semaphorins and their receptors lead to premature termination codon formation. Exon/intron summary of the new transcripts and experimental results are schematized in Figure 4. The newly identified *SEMA3C* transcript (accession no. LN626689) misses the exon 15. This splicing event leads to the formation of a premature stop codon in the exon 16 (Figure 4A). The new predicted semaphorin 3C encoded by this transcript is 237 amino acids shorter than the canonical protein and misses the Ig-like C2 type and the R/K rich domains (Figure S13). However, it still retains its Sema domain that is necessary for the homodimerization. The new *SEMA3C* transcript has a significantly lower expression in triple negative BC cell line than in non-tumor breast cell line, as depicted in Figure 4A.

**Figure 5:**
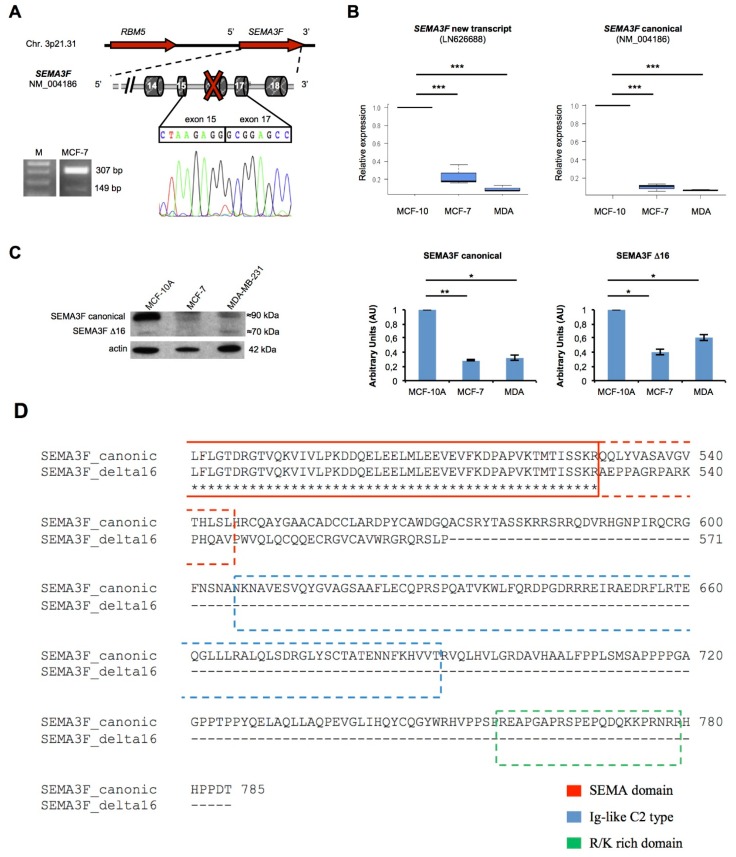
(**A**) Scheme of the new *SEMA3F* transcript and expression by RT-PCR on MCF-7 cell line, showing the canonical (307 bp) and the new alternative transcript (149 bp) of *SEMA3F*; M is 100 bp DNA marker; (**B**) Boxplots with the relative expression of both annotated and new *SEMA3F* transcript in breast cell lines measured with qRT-PCR (*n* = 3); (**C**) On the **left**, immunoreactive bands—corresponding to the canonical (about 90 kDa) and the new isoform (about 70 kDa) of semaphorin 3F—detected by Western Blot on whole breast cell lines lysates. On the **right**, the densitometry results (AU = Arbitrary Units) by ImageJ of the Western Blot bands, after normalization with actin, are shown as bar graphs (*n* = 2); (**D**) A detail of the protein alignment of the canonical and the new semaphorin 3F protein isoform is shown. The dashed boxes represent the functional domains that are deleted in the novel protein sequence. Asterisks indicate significant *p* values (* *p* < 0.05; ** *p* < 0.01; *** *p* < 0.001).

Conversely, the new transcript (accession no. LN626690) of plexin-B1 gene (*PLXNB1*) is highly expressed in BC cells, though differences compared to MCF-10A cell line are not significant (Figure 4B). Plexin-B1, the receptor for semaphorin 4D, is involved in axon guidance, invasive growth and cell migration through RhoA activation, with subsequent changes of the actin cytoskeleton [22]. Compared to the canonical protein, this new predicted isoform lacks 276 residues (position 1860–2135; Figure S14). However, no functional domains are affected, and we could not predict any function for this new plexin-B1 protein. Finally, a new transcript of *SEMA3F* gene, encoding semaphorin 3F protein, was discovered (Figure 5A), as discussed below.

### 2.5. A New Semaphorin 3F Isoform Expressed in Breast Cancer

Transcript-specific PCR assay and Sanger sequencing confirmed RNA-Seq data, *i.e.*, that the new transcript (accession no. LN626688) of *SEMA3F* gene is generated by exon 16 skipping. In addition, qRT-PCR analysis revealed both the canonical and the newly identified transcripts are down-modulated in the BC cells *vs.* non-tumoral cell line (Figure 5B). The expression of both *SEMA3F* transcripts was evaluated on a larger panel of human samples, revealing that these are not specifically expressed in breast tissues/cell lines (Figure S15).

In addition, the presence of two different immunoreactive bands of the expected sizes (about 89 and 72 kDa) in breast cell lines revealed that the new SEMA3F isoform is translated (Figure 5C). Protein densitometry confirmed the decreased expression of both SEMA3F isoforms in BC cell lines.

The new semaphorin 3F isoform shares residues 1–529 with the canonical protein and lacks 214 amino acids at the C-terminal (Figure 5D). However, as previously described for the sema 3C isoform, the newly identified sema 3F isoform lacks the Ig-like C2 type and the R/K rich domains, and still retains the Sema domain.

**Figure 6:**
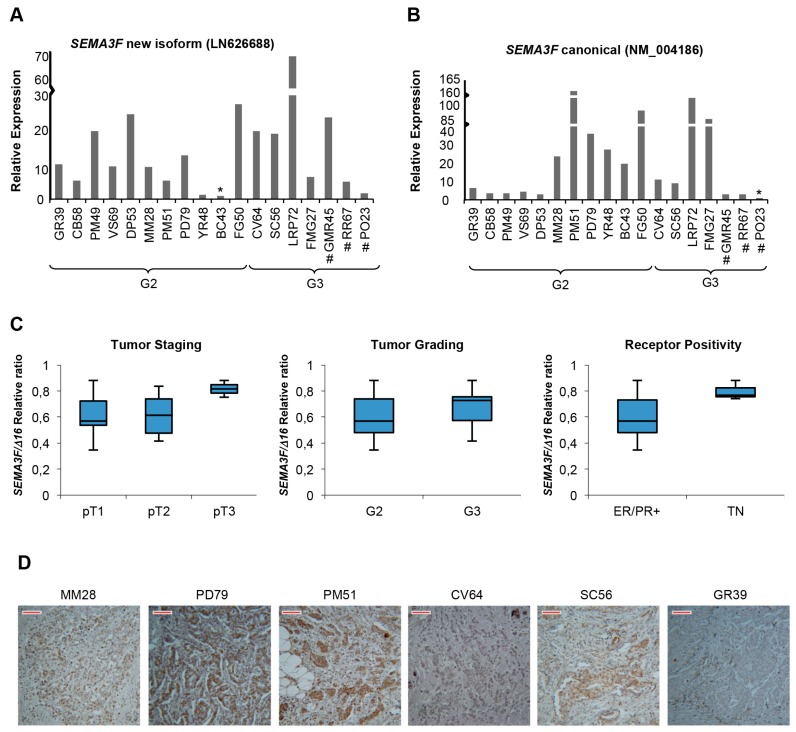
Semaphorin 3F expression in BC tumor biopsies (*n* = 18). (**A**,**B**) qRT-PCR analysis of the new (LN626688) and the annotated (NM_004186.3) *SEMA3F* transcripts, respectively. Samples have been ordered according to tumor grade (G2, G3); # indicates Triple Negative biopsies; asterisks (*) indicate the sample with the lowest absolute expression, used as reference; (**C**) Relative ratio of Δ*C*_t_ values between skipping and canonical mRNA isoforms of *SEMA3F* across breast cancer subtypes. TNM: pT1 (*n* = 7); pT2 (*n* = 9); pT3 (*n* = 2). Tumor grade: G2 (*n* = 11); G3 (*n* = 7). ER^+^/PR^+^ (*n* = 15); TN (triple negative samples) (*n* = 3); (**D**) Six representative images by immunohistochemistry assay on FFPE slices from breast cancer specimens are shown; N-terminal anti-Sema3F (that recognizes both the canonical and the new splicing variant) primary antibody was used. Scale Bar (in red) value = 33.76 µm.

Subsequently, we evaluated by end-point PCR and Sanger sequencing the presence of all the newly transcripts in human breast cancer specimens (*n* = 18). We found that although all the previously described new transcripts are expressed in human BC cells lines, they have a variable expression (about 10%–15% of the samples) in BC biopsies (data not shown). Interestingly, only *SEMA3F* (both the new and the canonical transcripts) are expressed in all analyzed tumor biopsies (Figure 6A,B). Quantitative analysis revealed a very heterogeneous expression among tumor biopsies. We could not find any correlation between the expression of both the new and the annotated *SEMA3F* isoforms—and specific clinical characteristics of BC (*i.e.*, subtypes, tumor stage, hormone receptors’ positivity). Moreover, to assess whether an imbalance between the two isoforms may exist across BC subtypes, we measured the relative ratio between *SEMA3F* canonical and new transcripts (Figure 6C). Again, we could not find any significant correlation.

Such high variability was also confirmed by immunohistochemistry performed on the same biopsies (examples in Figure 6D). Overall, a quite good mRNA/protein correlation was observed for analyzed samples.

## 3. Discussion

Solid tumors, including breast cancer, evolve toward metastatic spread of invasive cells [1]. Other than DNA mutations/rearrangements, AS can exert an oncogenic activity [23]. However, the impact of splicing in cancer is still underestimated. RNA-Sequencing is a powerful application of NGS that allows exploring cancer-related or -specific changes in gene expression and splicing patterns [7,24]. Splicing events occurring specifically or predominantly in cancer have been reported to have diagnostic/prognostic implications [25]. As the alteration of cell adhesion/motility is a hallmark of the metastatic process [4], we employed RNA-Seq to simultaneously investigate expression and alternative splicing in adhesion/motility-related genes in a specific estrogen-sensitive BC cells. Noteworthy, 12 new adhesion/motility-related and semaphorins/plexins transcripts were identified and experimentally validated. Computational predictions indicated that some of them (*THBS1*, *RHOA*, *RHOD*, *CASK*) encode proteins that lack important functional domains (description in Supplementary File and Figures S4–S14). However, the most relevant findings concerned the semaphorins/plexins axis. For instance, we found higher expression of the new transcript encoding plexin-B1 in triple negative metastatic BC cells *vs*. MCF-10 and MCF-7, suggesting a possible link with the metastatic phenotype (Figure 1C). Indeed, this protein is involved—through RhoA activation—in invasive growth and cell migration [22]. It can elicit multiple intracellular signals interacting with several cytoplasmic ligands and membrane tyrosin-kinase receptors, including MET, RHOD, NRP1 and NRP2. Mutagenesis in the cytoplasmic tail causes the loss of cytoskeleton remodeling in response to semaphorin 4D binding (UniProt). Notably, the new predicted plexin-B1 protein lacks 276 residues of the cytoplasmic tail. Therefore, it may lose the ability to interact with cytoplasmic effectors, possibly affecting microtubule remodeling and motility. However, this new transcript was expressed in less than 10% of BC tumor specimens. Further studies will help to clarify the real contribution—if any—of this new transcript in BC pathogenesis. Conversely, the new transcript of semaphorin 3F and also the canonical *SEMA3F* transcript are expressed in all BC biopsies. Immunohistochemistry revealed also its expression at the protein level. However, although we have detected an immunoreactive band corresponding to the expected size of the new SEMA3F protein isoform, we cannot exclude that—at least in part—the new isoform may be targeted by non sense-mediated decay (NMD), as the exon skipping causes the formation of a PTC located >50 nt upstream the last exon–exon junction of SEMA3F pre-mRNA.

Semaphorins, particularly classes 3 and 4, are involved in cancer progression [26,27], although conflicting results are reported [28]. Semaphorin 3F acts as an oncosuppressor, mainly through its anti-angiogenic properties [29] and the chemorepulsive action toward immune system cells [13]. Concordantly, it is down-regulated in many cancer types [30,31]. We experimentally confirmed that the new transcript identified by RNA-Seq encodes a new shorter semaphorin 3F isoform (Figure 5C). Sequence-based *in silico* analysis predicts it does not interact with neuropilins, but still retains the ability to form functional homodimers. These data suggest a potential dominant negative activity toward the canonical receptor. Further studies are needed to verify this hypothesis. Notably, we could not find any correlation between the expression of new SEMA3F isoform and tumor sbtype, stage and/or positivity to ER or PR. We are aware that the lack of correlation may be possibly due to the small number of analyzed samples.

Despite some of the newly discovered transcripts for adhesion/motility-related genes have low expression in tumor biopsies or they are expressed only in a small fraction of them, our results indicate that (1) genes relevant for cancer invasiveness are prone to alternative splicing; (2) these new transcripts may potentially encode proteins that lack functional domains and (3) gene annotations are still incomplete and need to be updated. Of note, our study did not reveal the presence of BC-specific isoforms. However, we cannot exclude that these isoforms undergo cancer-specific post-translational modifications or that they may define new protein–protein interactions networks, with a distinct role in BC cells, or that their presence in BC cells may confer selective advantage. BC cells are the central node of the tumor microenvironment, mainly constituted by tumor associated adipocytes and fibroblasts [32]. Chronic inflammatory milieu sustained by adipocyte-released factors is highly permissive for BC cells’ proliferation and metastasis. Again, we cannot exclude that rapidly growing BC cells—in the presence of alternative isoforms of cell–cell or cell–matrix interacting proteins—may differently communicate with surrounding cells, thus developing a more aggressive behavior. Functional studies will clarify the contribution of these new isoforms to the tumor microenvironment.

## 4. Experimental Section

### 4.1. Cell Lines and Tissue Samples

MCF-7 and MDA-MB-231 human BC cell lines were cultured, and BC specimen (*n* = 18) were collected, as described in a previous study [5], and the Research Project was approved (22 November 2010) by the Central Ethic Committee (CEC) at “Salvatore Maugeri” Foundation. All patients signed informed consent. Molecular subtypes and clinical data are reported in Table S1.

### 4.2. RNA Isolation and RNA-Sequencing

cDNA of human heart, liver, thyroid, monocytes, colon, kidney, adipocytes, EPC (endothelial progenitor cells), SVF (stromal vascular fraction cells), HEK293 and Jurkat cell lines was available in our laboratory. RNA from MCF-7 and MDA-MB-231 cell lines was isolated using TRIzol (Invitrogen, Carlsbad, CA, USA) according to manufacturer's instructions. MCF-10A RNA was kindly provided by Carmen Valente (IBP-CNR, Naples, Italy). Integrity was assessed by Experion (RNA StdSens Chip, Bio-Rad, Hercules, CA, USA). Total RNA was reverse transcribed using SuperScriptTM II Reverse transcriptase (Invitrogen) according to manufacturer’s protocol. cDNA libraries were prepared using TruSeqTM RNA Sample Preparation kit (Illumina, San Diego, CA, USA) as described elsewhere [33]. Paired-end reads were sequenced on Illumina HiSeq2000 platform. Mapping was performed using TopHat v2.0 (Figure S2). Gene expression and alternative splicing were analyzed using Cufflinks v2.1.1 [34] and our recently published pipeline [7]. The raw RNA-Seq data from this study are deposited in the NCBI Gene Expression Omnibus (GEO) database under accession number GSE68228.

### 4.3. In Silico Analysis

List of genes encoding cell-adhesion, -matrix and -motility genes were selected using the “Advanced search tool” of the GeneCards database (http://www.genecards.org; release October 2013). In detail, we specifically searched the terms “cell-adhesion”, “matrix” and “motility” to retrieve genes from literature and the major bioinformatics databases (as NCBI, Ensembl, SwissProt). New transcripts were identified by the procedure described in [9]. RNA-Seq results were inspected on UCSC Genome Browser (http://genome.ucsc.edu/cgi-bin/hgGateway). BLAT algorithm at UCSC was used for PCR products. ApE (http://biologylabs.utah.edu/jorgensen/wayned/ape/) was used to analyze electropherograms and to assess the presence of ORFs. Multiple sequence alignments were performed by ClustalW.

### 4.4. RT-PCR, Cloning and Sequencing

Semiquantitative RT-PCR assays using different PCR amplification cycles (25, 30, 35, 40) and different amount of starting cDNA templates have been performed to setup the experimental conditions for each primer pair (designed using Oligo 4.0 and listed in Table S2). Such approach was used to carry out a single end-point PCR assay per primer pair to simultaneously detect both the new splicing event and the canonical one.

End-point PCR assays with specific primer pairs were performed using MyTaq DNA Polymerase (Bioline, London, UK). PCR products for the characterization of the alternative splicing event were cloned into TopoVector II (Invitrogen), according to manufacturer’s instructions. Positive clones were sequenced by direct Sanger sequencing.

### 4.5. qRT PCR

qRT PCR was performed using iCycler iQ System (Bio-Rad, Hercules, CA, USA) and SYBR Green Supermix (Bio-Rad) according to manufacturer’s protocol. PPIA gene was used as reference. Samples were run in duplicates. Relative gene expression was measured using 2^−ΔΔ*C*t^ method. For BC tumor biopsies, the sample with the lowest expression was used as reference. Moreover, Δ*C*_t_ values of SEMA3F canonical and new transcript were used to calculate their relative ratio in each biopsy. Primers are listed in Table S2.

### 4.6. Western Blot and Immunohistochemistry

MCF-7 cells were washed with PBS, lysed on ice in NP-40 lysis buffer (0.5% NP-40, 120 mM NaCl, 50 mM Tris-HCl pH 8.0, 10 mM NaF, 1 mM Na_3_VO_4_, complete protease inhibitor cocktail) and centrifuged at 2500× *g* for 15 min at 4 °C. Cell culture supernatant was collected and centrifuged at 1500× *g* for 5 min. Protein preparation was performed as described in [5]. N-terminal anti-sema3F primary antibody (AB5471P, rabbit polyclonal, Merck Millipore, Billerica, MA, USA) was used. For total protein normalization, we used anti-actin primary antibody (A2066, rabbit polyclonal, Sigma-Aldrich, Saint Louis, MO, USA). FFPE slices from each cancer sample along with control were processed for sema3F staining (SAB2700501, rabbit polyclonal, Sigma-Aldrich). Epitope retrieval was performed in pre-warmed TE buffer, pH 9 (Dako, Glostrup, Denmark) for 40 s at 98 °C. Incubation with anti-sema3F was carried out at room temperature for 30 s. Antigen detection was performed using LSAB-Plus/HRP kit (Dako). Nuclei were counterstained with haematoxylin. Analysis was carried out with a DM1000 Microscope (Leica, Wetzlar, Germany ) equipped with LAS Software (Leica) for images capture. Semaphorin 3F localization was evaluated as percentage of positive cells (Low <10%, Moderate 10%–50%, High >50%). Example images (fifty randomly selected regions) are displayed at 200× magnification.

### 4.7. Statistics

PCR amplicons from semi-quantitative RT-PCR were run on 1%–2% agarose gels. For each newly identified isoform, PCR bands were quantified using ImageJ software (National Institutes of Health, Bethesda, MD, USA). In particular, pixel density of each PCR gel band has been compared to pixel density of a DNA marker of known concentration (Low Ranger 100 bp DNA Ladder, Norgen, Thorold, ON, Canada) to normalize data across replicates and cell lines. Pixel ratio (or Arbitrary Unit) were plotted in boxplots. Data was analyzed by one-way analysis of variance (ANOVA) using the open statistical computing language R. In every ANOVA, Tukey’s Honestly Significant Difference pair-wise comparison test was used as *post*-*hoc* test for multiple comparison. *p*-values of less than 0.05 were considered to be significant. Paired *t* test was used to analyze Western blot quantification data.

## Supplementary Materials

Supplementary materials can be found at http://www.mdpi.com/1422-0067/17/1/121/s1.

## References

[1] Ferlay J., Soerjomataram I., Ervik M., Dikshit R., Eser S., Mathers C., Rebelo M., Parkin D.M., Forman D., Bray F. GLOBOCAN 2012: Estimated Cancer Incidence, Mortality and Prevalence Worldwide in 2012. http://globocan.iarc.fr.

[2] Assinder S.J., Au E., Dong Q., Winnick C. (2010). A novel splice variant of the β-tropomyosin (*TPM2*) gene in prostate cancer. Mol. Carcinog..

[3] Valastyan S., Weinberg R.A. (2011). Tumor metastasis: Molecular insights and evolving paradigms. Cell.

[4] Palmer T.D., Ashby W.J., Lewis J.D., Zijlstra A. (2011). Targeting tumor cell motility to prevent metastasis. Adv. Drug Deliv. Rev..

[5] D’Apice L., Costa V., Valente C., Trovato M., Pagani A., Manera S., Regolo L., Zambelli A., Ciccodicola A., de Berardinis P. (2013). Analysis of *SEMA6B* gene expression in breast cancer: Identification of a new isoform. Biochim. Biophys. Acta.

[6] Pal S., Gupta R., Davuluri R.V. (2012). Alternative transcription and alternative splicing in cancer. Pharmacol. Ther..

[7] Eswaran J., Horvath A., Godbole S., Reddy S.D., Mudvari P., Ohshiro K., Cyanam D., Nair S., Fuqua S.A., Polyak K. (2013). RNA sequencing of cancer reveals novel splicing alterations. Sci. Rep..

[8] Liu J., Lee W., Jiang Z., Chen Z., Jhunjhunwala S., Haverty P.M., Gnad F., Guan Y., Gilbert H.N., Stinson J. (2012). Genome and transcriptome sequencing of lung cancers reveal diverse mutational and splicing events. Genome Res..

[9] Scarpato M., Esposito R., Evangelista D., Aprile M., Ambrosio M.R., Angelini C., Ciccodicola A., Costa V. (2014). AnaLysis of Expression on human chromosome 21, ALE-HSA21: A pilot integrated web resource. Database (Oxford).

[10] Chekhun S., Bezdenezhnykh N., Shvets J., Lukianova N. (2013). Expression of biomarkers related to cell adhesion, metastasis and invasion of breast cancer cell lines of different molecular subtype. Exp. Oncol..

[11] Worzfeld T., Swiercz J., Looso M., Straub B.K., Sivaraj K.K., Offermanns S. (2012). ErbB-2 signals through Plexin-B1 to promote breast cancer metastasis. J. Clin. Investig..

[12] Miyato H., Tsuno N.H., Kitayama J. (2012). Semaphorin 3C is involved in the progression of gastric cancer. Cancer Sci..

[13] Mendes-da-Cruz D.A., Brignier A.C., Asnafi V., Baleydier F., Messias C.V., Lepelletier Y., Bedjaoui N., Renand A., Smaniotto S., Canioni D. (2014). Semaphorin SEMA3F and Neuropilin-2 control the migration of human T-cell precursors. PLoS ONE.

[14] Goldblum S.E., Young B.A., Wang P., Murphy-Ullrich J.E. (1999). Thrombospondin-1 induces tyrosine phosphorylation of adherens junction proteins and regulates an endothelial paracellular pathway. Mol. Biol. Cell.

[15] Zheng H., Gao L., Feng Y., Yuan L., Zhao H., Cornelius L.A. (2009). Down-regulation of Rap1GAP via promoter hypermethylation promotes melanoma cell proliferation, survival, and migration. Cancer Res..

[16] Elola M.T., Wolfenstein-Todel C., Troncoso M.F., Vasta G.R., Rabinovich G.A. (2007). Galectins: Matricellular glycan-binding proteins linking cell adhesion, migration, and survival. Cell. Mol. Life Sci..

[17] Reinhold M.I., Lindberg F.P., Plas D., Reynolds S., Peters M.G., Brown E.J. (1995). *In vivo* expression of alternatively spliced forms of integrin-associated protein (CD47). J. Cell Sci..

[18] Sadok A., Marshall C. (2014). Rho GTPases: Masters of cell migration. Small GTPases.

[19] Hsueh Y. (2009). Calcium/calmodulin-dependent serine protein kinase and mental retardation. Ann. Neurol..

[20] Xing F., Okuda H., Watabe M., Kobayashi A., Pai S.K., Liu W., Pandey P.R., Fukuda K., Hirota S., Sugai T. (2011). Hypoxia-induced Jagged2 promotes breast cancer metastasis and self-renewal of cancer stem-like cells. Oncogene.

[21] Van Rossum A.G., de Graaf J.H., Schuuring-Scholtes E., Kluin P.M., Fan Y.X., Zhan X., Moolenaar W.H., Schuuring E. (2003). Alternative splicing of the actin binding domain of human cortactin affects cell migration. J. Biol. Chem..

[22] Ch’ng E.S., Kumanogoh A. (2010). Roles of Sema4D and Plexin-B1 in tumor progression. Mol. Cancer.

[23] David C.J., Manley J.L. (2010). Alternative pre-mRNA splicing regulation in cancer: Pathways and programs unhinged. Genes Dev..

[24] Wu Y., Wang X., Wu F., Huang R., Xue F., Liang G., Tao M., Cai P., Huang Y. (2012). Transcriptome profiling of the cancer, adjacent non-tumor and distant normal tissues from a colorectal cancer patient by deep sequencing. PLoS ONE.

[25] Thorsen K., Sorensen K.D., Brems-Eskildsen A.S., Modin C., Gaustadnes M., Hein A.M., Kruhøffer M., Laurberg S., Borre M., Wang K. (2008). Alternative splicing in colon, bladder, and prostate cancer identified by exon array analysis. Mol. Cell. Proteom..

[26] Sakurai A., Doci C.L., Gutkind J.S. (2012). Semaphorins signaling in angiogenesis, lymphangiogenesis and cancer. Cell Res..

[27] Tamagnone L. (2012). Emerging role of Semaphorins as major regulatory signals and potential therapeutic targets in cancer. Cancer Cell.

[28] Swiercz J., Worzfeld T., Offermans S. (2007). ErbB-2 and met reciprocally regulate cellular signaling via plexin-B1. J. Biol. Chem..

[29] Parker M.W., Hellman L.M., Xu P., Fried M.G., Vander Kooi C.W. (2010). Furin processing of Semaphorin3F determines its anti-angiogenic activity by regulating direct binding and competition for neuropilin. Biochemistry.

[30] Doçi C.L., Mikelis C.M., Lionakis M.S., Molinolo A.A., Gutkind J.S. (2015). Genetic identification of SEMA3F as an antilymphangiogenic metastasis suppressor gene in head and neck squamous carcinoma. Cancer Res..

[31] Xiong G., Wang C., Evers B.M., Zhou B.P., Xu R. (2012). RORα suppresses breast tumor invasion by inducing SEMA3F expression. Cancer Res..

[32] Quail D.F., Joyce J.A. (2013). Microenvironmental regulation of tumor progression and metastasis. Nat. Med..

[33] Costa V., Esposito R., Ziviello C., Sepe R., Bim L.V., Cacciola N.A., Decaussin-Petrucci M., Pallante P., Fusco A., Ciccodicola A. (2015). New somatic mutations and WNK1-B4GALNT3 gene fusion in papillary thyroid carcinoma. Oncotarget.

[34] Trapnell C., Roberts A., Goff L., Pertea G., Kim D., Kelley D.R., Pimentel H., Salzberg S.L., Rinn J.L., Pachter L. (2012). Differential gene and transcript expression analysis of RNA-Seq experiments with TopHat and Cufflinks. Nat. Protoc..

