# Supplementary material for: Alternative Splicing in Adhesion- and Motility-Related Genes in Breast Cancer
Source: Int J Mol Sci. 2016 Jan 16;17(1):121. doi: 10.3390/ijms17010121 (PMC4730362; doi:10.3390/ijms17010121)
Supplement: Supplementary file 1 [file ijms-17-00121-s001.pdf]

# Supplementary Materials: Alternative Splicing in Adhesion- and Motility-Related Genes in Breast Cancer

**Rosanna Aversa, Anna Sorrentino, Roberta Esposito, Maria Rosaria Ambrosio, Angela Amato, Alberto Zambelli, Alfredo Ciccodicola, Luciana D'Apice and Valerio Costa**

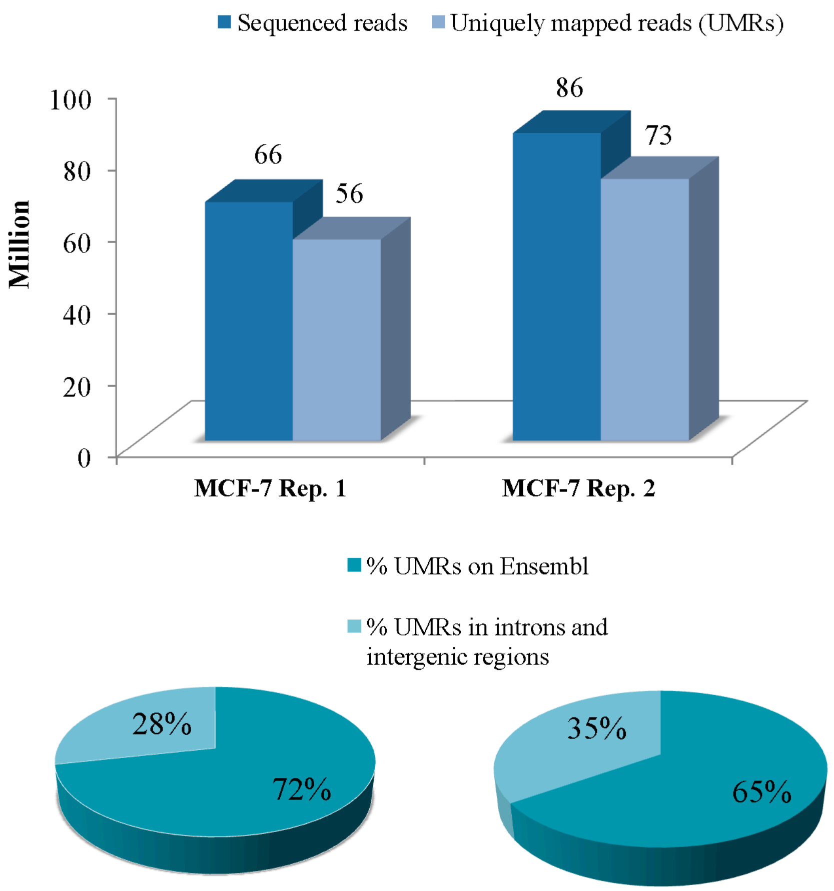


**Figure S1.** RNA-Sequencing mapping summary.

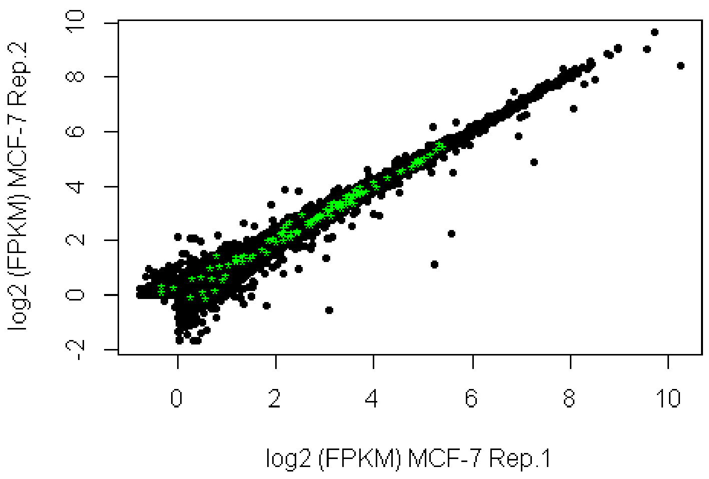


(**A**)

**Figure S2.** *Cont.*

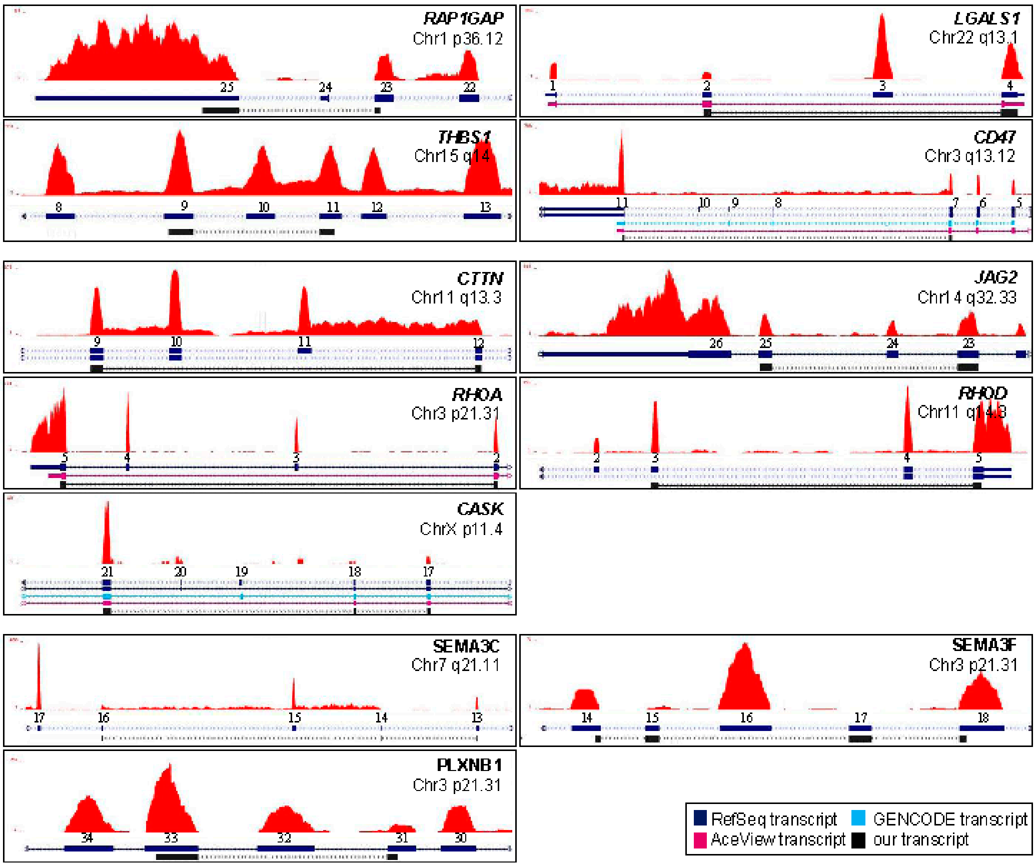


(**B**)

**Figure S2**. Gene expression analysis by RNA-Seq. (**A**) Correlation plot of normalized gene expression values (RNA-Seq) in MCF-7 replicates; green asterisks indicate genes encoding adhesion/ motility-related molecules, semaphorins and their receptors; (**B**) Screenshot of UCSC Genome Browser Session containing expression track of our RNA-Seq experiment. Blue boxes indicate the annotated (RefSeq or GENCODE) exons; pink boxes correspond to the AceView annotation; black boxes indicate newly identified splice junctions. Red peaks represent the coverage of mapped reads.

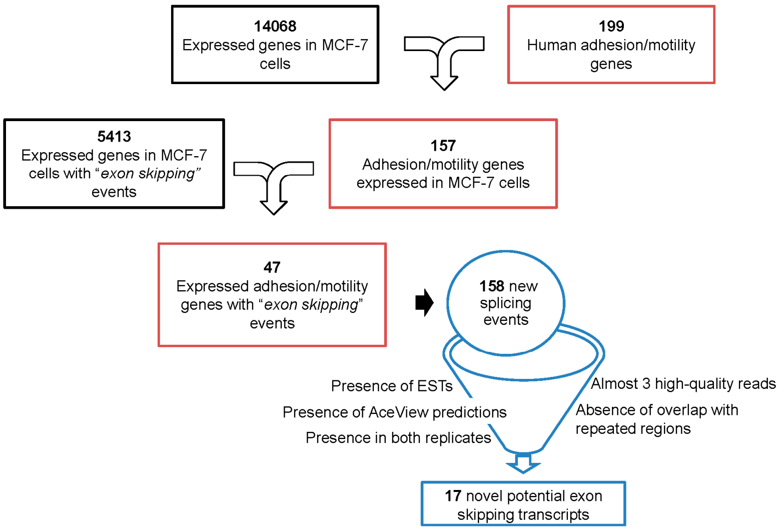


**Figure S3.** Schematic representation of the computational workflow for the identification of novel potential transcripts generated by alternative splicing. Genes with a FPKM higher than 1 were considered "expressed". Human adhesion/motility-related genes were retrieved from GeneCards database, and a customized procedure was used for the prediction of new exon skipping events.

**Table S1.** Clinical/histological characteristics of breast cancer specimens.

| ID | ER% | PR% | Her2 | Ki67 | Grade | TNM |
|---|---|---|---|---|---|---|
| BC43 | 90 | 90 | 1 | 15 | G2 | pT1c |
| CB58 | 80 | 90 | 1 | 15 | G2 | pT1c |
| CV64 | 80 | 70 | 1 | 8 | G2 | pTy3 |
| DP53 | 0 | 0 | 0 | 30 | G3 | pT2 |
| FG50 | 80 | 80 | 0 | 10 | G3 | pT2 |
| FMG27 | 90 | 90 | 0 | 15 | G2 | pT2 |
| GMR45 | 80 | 90 | 0 | 15 | G2 | pTy3 |
| GR39 | 90 | 90 | 0 | 10 | G2 | pT2 |
| LRP72 | 90 | 90 | 0 | 70 | G3 | pT1c |
| MM28 | 80 | 80 | 1 | 8 | G2 | pT1 |
| PD79 | 80 | 30 | 1 | 50 | G3 | pT2 |
| PM49 | 25 | 0 | 3 | 30 | G3 | pT1c |
| PM51 | 90 | 70 | 0 | 10 | G2 | pT1c |
| PO23 | 90 | 80 | 0 | 10 | G2 | pT2 |
| RR67 | 0 | 0 | 0 | 80 | G3 | pT1c |
| SC56 | 80 | 70 | 1 | 5 | G2 | pT2 |
| VS69 | 90 | 60 | 0 | 30 | G2 | pT1b |
| YR48 | 90 | 90 | 1 | 15 | G3 | pT2 |

ER/PR: Estrogen and Progesterone Receptors; Her2: Human Epidermal growth factor Receptor 2; T: Tumor size; N: lymph Nodes; M: metastasis.

**Table S2.** Primer pairs used for validation and quantification by qRT-PCR of alternative spliced transcript variants.

| Gene | Primer Sequences End Point PCR | | Amplicons (bp) | |
|---|---|---|---|---|
| | Forward 5′-3′ | Reverse 5′-3′ | canonic | New |
| *SEMA3C* | GGAGGAGCTGGAAGTCTTTA | GCATTGAGTCAGTGGGTTTC | 271 | 113 |
| *SEMA3F* | AGGTGGAGGTCTTCAAGGAT | GACTCCACGGCATTCTTGTT | 307 | 149 |
| *PLXNB1* | CGGGAAAACCAGGATTATGT | TCGGAGATGCCATGCTGCT | 332 | 159 |
| *LGALS1* | GCAACCTGAATCTCAAACCT | TCAGTCAAAGGCCACACATT | 386 | 214 |
| *RHOD* | GCAAGATGACTATGACCGCC | TCAGGTCACCACGCAAAAGC | 412 | 277 |
| *THBS1* | GTCCCCGTGGTCATCTTGTT | AGGGGGACAAGCACCACATT | 409 | 235 |
| *JAG2* | TGCGGATGGAAGCCTTGTCT | CGTAACAACCGTCTCCACCT | 510 | 378 |
| *CASK* | AATCAATGGCATCAGTGTGG | TGAGACCTGCAGTTCCATTT | 401, 365, 332 | 296 |
| *RAP1GAP* | TGGAAGCATCTGAGCAGCAC | CGATGCCAGCAGCTTCCCT | 264 | 221 |
| *CTTN* | TCCAAAGGTTTCGGCGGCAA | TCCATCCGATCCTTCTGCAC | 380, 269 | 158 |
| *RHOA* | TGGTGATTGTTGGTGATGGA | AGCACTTCAAAATTAACCGCA | 606 | 354 |
| *CD47* | TCTTAGCTCTAGCACAATTAC | TTTTCTTGTTTCTTCTCCCCA | 257, 224, 199 | 167 |
| | Primer Sequences qRT-PCR | | | |
| *SEMA3FΔ16* | ACCATCTCTTCTAAGAGGGC | | 110 | |
| CTRL + | ACCATCTCTTCTAAGAGGCA | GACTCCACGGCATTCTTGTT | 266 | |
| CTRL - | ACCATCTCTTCTAAGAGGTC | | - | |
| *SEMA3F* | AGGTGGAGGTCTTCAAGGAT | TTGGAGGATGCTGTATAGCG | 214 | |

## Adhesion-related genes

Using primer pairs within exons 9 and 11 of *THBS1* RefSeq annotated transcript (NM_003246.2), two PCR products of 409 and 235 bp were amplified. These amplicons were then sequenced by Sanger method. BLAST analysis revealed that the longer amplicon matches to the RefSeq annotated transcript, whereas the shorter one corresponds to the new AS transcript, as indicated by RNA-Seq analysis. The newly identified exon skipping event is not reported in any *THBS1* transcript annotated in RefSeq, Ensembl and GENCODE databases, nor in AceView and EST databases. The protein encoded by *THBS1* gene is a subunit of a disulfide-linked homotrimeric protein, which is an adhesive glycoprotein that mediates cell-to-cell and cell-to-matrix interactions. The annotated thrombospondin-1 (UniProtID: P07996) is a multi-domain protein, 1170 amino acids long, characterized by a von

Willebrand Factor C domain, three TSP type 1 domains, two EGF like domains, five TSP type 3 domains and a heparin binding region. The novel identified *THBS1* transcript is predicted to encode a shorter protein that consists of 1112 amino acids. ClustalW2 alignment revealed that the novel putative isoform shares the amino acids from 1 to 428 and from 550 to 1170 with the annotated thrombospondin-1 protein. This *in silico* analysis also revealed that residues missing in the novel isoform correspond to two of the three TSP type 1 domains, known to inhibit angiogenesis and endothelial cell migration. In addition, the new thrombospondin-1 protein lacks two amino acids of an EGF-like domain of canonical THBS1 protein (Figure S4).

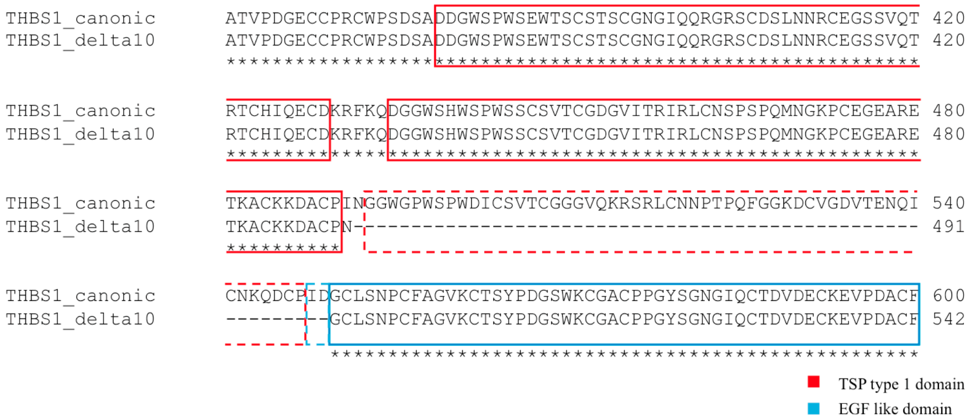


**Figure S4.** New THBS1 protein alignment. A detail of the protein alignment of the canonical and the new thrombospondin-1 protein isoform is shown. The dashed boxes represent the functional domains that are deleted in the novel protein sequence.

The skipping of exon 24 in *RAP1GAP* gene was revealed by a 221 bp PCR product, shorter than the RefSeq annotated transcript (264 bp). The splicing event leads to the loss of the canonic STOP codon and the formation of a new one within exon 25, corresponding to the 3′UTR region of the annotated transcript. The new predicted ORF for *RAP1GAP* consists of 2466 bp, whereas the canonical ORF is 2184 bp long. Rap1GAP protein can inhibit cell proliferation and migration, acting as a specific negative regulator of Rap1, which is known to promote melanoma cell proliferation and migration through the mitogen-activated protein kinase pathway and integrin activation [15]. The putative encoded protein consists of 821 amino acids, whereas the annotated one (UniProt ID: P47736) is 727 aa long, displaying additional 94 amino acids at the C-terminus (Figure S5). BLASTP analysis revealed that the novel 96 residues are evolutionary conserved among mammals (not shown). The evolutionarily conservation of this sequence suggests that the new 96 amino acids could be part of a domain with a functional role. However, the lack of annotated similar protein structures on PDB database has hampered the prediction of the tridimensional structure of the new putative domain.

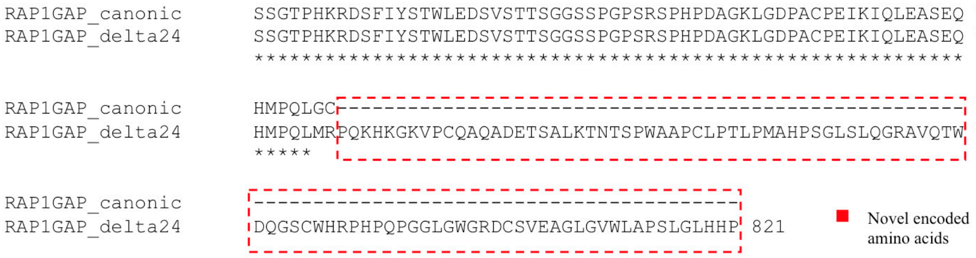


**Figure S5.** New RAP1GAP protein alignment. A detail of the protein alignment of the canonical and the new rap1GAP protein isoform is shown. The dashed box represents the novel amino acids that are encoded by the novel sequence.

The exon skipping identified by RNA-Seq for the *LGALS1* gene determines frame-shift with the formation of PTC in position 389 of the annotated transcript (RefSeq: NM_002305.3). RT-PCR assay showed the presence of an amplicon of 214 bp, other than the canonic product (386 bp). Sequencing of the shorter PCR product and BLAST analysis revealed the lack of 172 bp compared to the RefSeq *LGALS1* transcript, corresponding to the skipping of exon 3. The new transcript overlaps six ESTs (BQ276726, BU957899, BG500128, BQ276759, BU587948 and BF185924) and is supported by the AceView gene prediction LGALS1.gAug10. Galectin-1 (Gal-1), the protein product of *LGALS1* gene, is an evolutionarily conserved β-galactoside-binding lectin that regulates endothelial cell migration, proliferation and adhesion, with a key role in tumor-immune escape, tumor growth and metastasis. The AS event identified by RNA-Seq and further validated in *LGALS1* leads to PTC formation, 116 bp upstream the canonical STOP codon. Hence, the new protein isoform is predicted to undergo NMD-mediated degradation. However, if translated, the putative encoded protein would consist of only 39 amino acids, and would lack both the β-galactoside binding regions (Figure S6).

```
LGALS1_canonic     MACGLVASNLNLKPGECLRVRGEVAPDAKSFVLNLGKDSNNLCLHFNPRFNAHGDANTIV 60
LGALS1_delta3      MACGLVASNLNLKPGECLRVRGEVAPDAKRCASPSTRPT--------------------- 39
                   *****************************

LGALS1_canonic     CNSKDGGAWGTEQREAVFPFQPGSVAEVCITFDQANLTVKLPDGYEFKFPNRLNLEAINY 120
LGALS1_delta3      ------------------------------------------------------------

LGALS1_canonic     MAADGDFKIKCVAFD 135
LGALS1_delta3      ---------------
```

β-galactoside binding site

**Figure S6.** New LGALS1 protein alignment. Protein alignment of the canonical and the new galectin-1 protein isoform is shown. The dashed boxes represent the functional domains that are deleted in the novel protein sequence.

Finally, using primer pairs within exons 7 and 11 of *CD47* RefSeq annotated transcript 1 (NM_001777.3), we obtained four PCR products of 257 bp, 224 bp, 199 bp and 167 bp, respectively. Cloning and further Sanger sequencing of these amplicons revealed that the longer PCR product corresponds to RefSeq variant 1 (NM_001777.3), the two intermediate products (224 bp and 199 bp) correspond to Ensembl transcript ENST00000355354 and RefSeq transcript 2 (NM_198793.2) respectively, while the shorter one (167 bp) corresponds to a new transcript generated by AS of exons 8, 9 and 10, as predicted by RNA-Seq data. The spliced transcript overlaps thirteen ESTs (AA418701, AI167998, BM978478, HY266402, BF792592, BF792654, DB366420, AA418709, BF034098, AL520436, BF996233, AA478755 and BE094468) and is supported by the AceView gene prediction CD47.bAug10. CD47, also known as Integrin-Associated Protein (IAP), is a receptor for thrombospondin family members, a ligand for the transmembrane signaling protein *SIRPα* and a component of a supramolecular complex containing specific integrins, heterotrimeric G proteins and cholesterol. CD47 has a role in cell adhesion, cell proliferation, apoptosis and angiogenesis, by acting as an adhesion receptor for thrombospondin1, and is an important regulator of integrin function. This glycoprotein is a 50 kDa plasma membrane protein, 323 aa long, with an amino-terminal extracellular sequence consisting of a single immunoglobulin domain (IgV), five transmembrane-spanning domains and a short alternatively spliced intracytoplasmic tail. In both humans and mice, the cytoplasmic tail can be found as four different splice isoforms ranging from 4 to 34 amino acids, showing different tissue expression patterns [17]. The new *CD47* mRNA variant, which lacks exons 8, 9 and 10, encodes a predicted protein of 293 residues with a 4 amino acids long cytoplasmic tail (Figure S7).

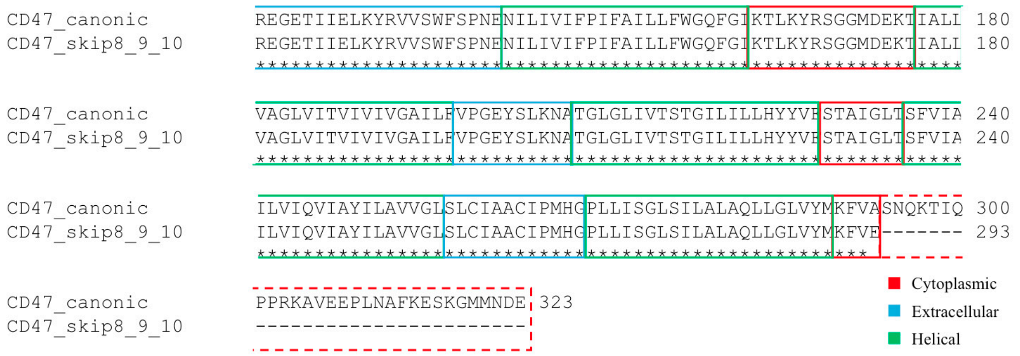


**Figure S7.** New CD47 protein alignment. A detail of the protein alignment of the canonical and the new CD47 protein isoform is shown. The dashed boxes represent the functional domains that are deleted in the novel protein sequence.

## Motility-related genes

RT-PCR assay detected for *RHOA* gene two amplicons of different length (606 bp and 354 bp), the former corresponding to the RefSeq annotated mRNA isoform (NM_001664.2), the latter to the new transcript that skips exons 3 and 4. Sanger sequencing further confirmed the nature of the new transcript. *In silico* analysis revealed that the spliced sequence is also supported by the presence of a gene prediction reported in AceView (RHOA.kAug10) and by the presence of four ESTs (BQ231766, BG168382, CD357891, CX756429) covering the splice junction. Similarly, for *RHOD* gene, we detected a PCR product of 277 bp, other than a 412 bp amplicon corresponding to the RefSeq canonic transcript (NM_014578.3). Sequencing of the new transcript revealed the presence of a skipping event for the exon 4. Even if no evidences from public databases support the presence of this sequence, we were able to *in vitro* confirm the *bona fide* of this new transcript identified by RNA-Seq. RhoA and RhoD are two small (~21 kD) GTPase protein, characterized by three GTP binding domains. Rho GTPases are key operators in signalling transduction pathways that control cell behaviour in response to signals from the extracellular environment [18]. The newly identified *RHOA* transcript is predicted to encode a protein of 109 aa. Its alignment with the annotated RhoA protein (UniProt ID: P61586), which consists of 193 aa, reveals they share amino acids 1-52 and 135-193. Thus, the new predicted RhoA protein lacks amino acids from 53 to 134, which form two GTP binding sites (Figure S8). Likewise, the new *RHOD* transcript, which skips exon 4, is predicted to encode a protein of 165 amino acids, missing residues 110 to 154 of the annotated protein (UniProt ID: O00212) that is 210 aa long. RhoD novel isoform maintains two out of three GTP binding sites of the canonic sequence, while loses the GTP binding site in positions 129-132 (Figure S9).

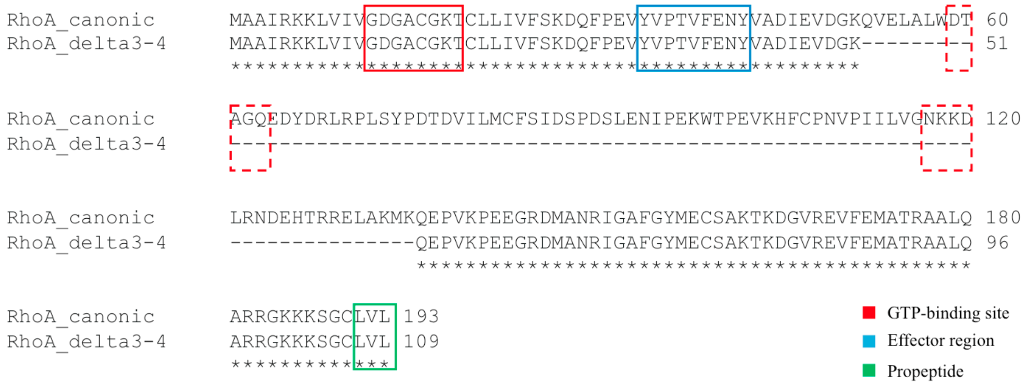


**Figure S8.** New RHOA protein alignment. Protein alignment of the canonical and the new RhoA protein isoform is shown. The dashed boxes represent the functional domains that are deleted in the novel protein sequence.

```
RhoD_canonic      MTAAQAAGEEAPPGVRSVKVVLVGDGGCGKTSLLMVFADGAFPESYTPTVFERYMVNLQV 60
RhoD_delta4       MTAAQAAGEEAPPGVRSVKVVLVGDGGCGKTSLLMVFADGAFPESYTPTVFERYMVNLQV 60
                  ************************************************************

RhoD_canonic      KGKPVHLHIWDTAGQDDYDRLRPLFYPDASVLLLCFDVTSPNSFDNIFNRWYPEVNHFCK 120
RhoD_delta4       KGKPVHLHIWDTAGQDDYDRLRPLFYPDASVLLLCFDVTSPNSFDNIFN----------- 109
                  *************************************************

RhoD_canonic      KVPIIVVGCKTDLRKDKSLVNKLRRNGLEPVTYHRGQEMARSVGAVAYLECSARLHDNVH 180
RhoD_delta4       ----------------------------------RGQEMARSVGAVAYLECSARLHDNVH 135
                                                    **************************

RhoD_canonic      AVFQEAAEVALSSRGRNFWRRITQGFCVVT 210
RhoD_delta4       AVFQEAAEVALSSRGRNFWRRITQGFCVVT 165
                  ******************************
```

GTP-binding site
Effector region
Propeptide

**Figure S9.** New RHOD protein alignment. Protein alignment of the canonical and the new RhoD protein isoform is shown. The dashed box represents the functional domain that is deleted in the novel protein sequence.

For *CASK* gene, the validation of the transcript that skips the exons 19 and 20 was revealed by the presence of a 296 bp PCR product, confirmed by Sanger sequencing approach. RT-PCR assay showed the presence of other three amplicons (401, 365 and 332 bp, respectively), deriving from already annotated RefSeq and GENCODE mRNA isoforms. In particular, the PCR product 401 bp long corresponded to the RefSeq transcript variant 1 (NM_003688.3); the 365 bp amplicon to GENCODE transcript that lacks the exon 20 of RefSeq variant 1, whereas the 332 bp product corresponds to a transcript missing only the exon 19, annotated in RefSeq transcript variants 2 and 3 (NM_001126054.2, NM_001126055.2). The new transcript overlaps an AceView gene prediction (CASK.gAug10) and the EST BP382934. *CASK* gene encodes for the Calcium/calmodulin-dependent serine protein kinase (CASK), a protein that belongs to the membrane-associated guanylate kinase protein family. The members of this family function as multiple domain adaptor proteins originally identified at cell junctions and synapses [19]. CASK protein is characterized by a protein kinase domain, two L27 domains, one PDZ and one SH3 domain, and a guanylate kinase-like domain at C-terminal part. The new putative protein of CASK consists of 886 residues, whereas the annotated protein is 921 aa long (UniProt ID: Q14247). The amino acids that are lost in the new isoform (580-614) are part of a linker region between the PDZ domain and the SH3 domain (Figure S10). The translation of this new spliced transcript is supported, other than the presence of ORF, also by the presence in UniProt database of other isoforms (IDs: O14936-3, O14936-4, O14936-5), which lack aa 580-602 (corresponding to the first part of the linker region), and of the isoform O14936-6 that lacks the aa 603-614, corresponding to the second part of the linker region.

```
CASK_canonic       DMENVTRVRLVQFQKNTDEPMGITLKMNELNHCIVARIMHGGMIHRQGTLHVGDEIREIN 540
CASK_delta19-20    DMENVTRVRLVQFQKNTDEPMGITLKMNELNHCIVARIMHGGMIHRQGTLHVGDEIREIN 540
                   ************************************************************

CASK_canonic       GISVANQTVEQLQKMLREMRGSITFKIVPSYRTQSSSCERDSPSTSRQSPANGHSSTNNS 600
CASK_delta19-20    GISVANQTVEQLQKMLREMRGSITFKIVPSYRTQSSSCE--------------------- 579
                   ***************************************

CASK_canonic       VSDLPSTTQPKGRQIYVRAQFEYDPAKDDLIPCKEAGIRFRVGDIIQIISKDDHNWWQGK 660
CASK_delta19-20    --------------IYVRAQFEYDPAKDDLIPCKEAGIRFRVGDIIQIISKDDHNWWQGK 625
                                 **********************************************

CASK_canonic       LENSKNGTAGLIPSPELQEWRVACIAMEKTKQEQQASCTWFGKKKKQYKDKYLAKHNADL 720
CASK_delta19-20    LENSKNGTAGLIPSPELQEWRVACIAMEKTKQEQQASCTWFGKKKKQYKDKYLAKHNADL 685
                   ************************************************************
```

PDZ domain
SH3 like domain

**Figure S10.** New CASK protein alignment. A detail of the protein alignment of the canonical and the new CASK protein isoform is shown.

RT-PCR assay for *JAG2* revealed two products of 510 bp and 378 bp, respectively. Direct Sanger sequencing and a BLAST analysis confirmed that shorter amplicon corresponds to a new AS transcript lacking the exon 24, compared to the annotated sequence (RefSeq: NM_002226.4). Jagged-2, the protein encoded by *JAG2* gene, is one of the ligands of Notch receptor. In mammals, their function is mostly linked to transduction signaling during development. However, it has been shown that mammalian Notch family members also possess the function of cell adhesion molecules. There are evidences that the expression of Jagged-2 is strongly upregulated at the hypoxic invasive front in breast cancers samples [20]. Jagged-2 is a transmembrane protein, with the N-terminal residues (27–1080) that are extracellular. Jagged-2 novel encoded protein is predicted to share with the annotated protein (UniProt ID: Q9Y219) residues 1 to 984 and 1029 to 1238. Most of the missing amino acids don't belong to any functional annotated domain, so we cannot predict any effect on protein functionality. It could be relevant to note, however, that residues 984-994, which are absent in the novel putative isoform, belong to Von Willebrand Factor C (VWFC) domain (Figure S11).

```
JAG2_canonic      CSKVWCGWKPCLLAGQPEALSAQCPLGQRCLEKAPGQCLRPPCEAWGECGAEEPPSTPCL 960
JAG2_delta24      CSKVWCGWKPCLLAGQPEALSAQCPLGQRCLEKAPGQCLRPPCEAWGECGAEEPPSTPCL 960
                  ************************************************************

JAG2_canonic      PRSGHLDNNCARLTLHFNRDHVPQGTTVGAICSGIRSLPATRAVARDRLLVLLCDRASSG 1020
JAG2_delta24      PRSGHLDNNCARLTLHFNRDHVPQ------------------------------------ 984
                  ************************

JAG2_canonic      ASAVEVAVSFSPARDLPDSSLIQGAAHAIVAAITQRGNSSLLLAVTEVKVETVVTGGSST 1080
JAG2_delta24      --------SFSPARDLPDSSLIQGAAHAIVAAITQRGNSSLLLAVTEVKVETVVTGGSST 1036
                          ****************************************************

JAG2_canonic      GLLVPVLCGAFSVLWLACVVLCVWWTRKRRKERERSRLPREESANNQWAPLNPIRNPIER 1140
JAG2_delta24      GLLVPVLCGAFSVLWLACVVLCVWWTRKRRKERERSRLPREESANNQWAPLNPIRNPIER
                  ************************************************************
```

VWFC domain
TM domain

**Figure S11.** New JAG2 protein alignment. A detail of the protein alignment of the canonical and the new jagged-2 protein isoform is shown. The dashed box represents part of the functional domain that is deleted in the novel protein sequence.

Using primer pairs within exons 9 and 12 of *CTTN* RefSeq annotated transcript (NM_005231.3), we obtained three PCR products of 380 bp, 269 and 158 bp, respectively. Cloning and further Sanger sequencing of these amplicons revealed that the longer PCR product corresponds to RefSeq variant 1 (NM_005231.3), the intermediate (269 bp) to RefSeq transcripts 2 and 3 (NM_138565.2 and NM_001184740.1), and the shorter one (158 bp) corresponds to a new transcript generated by AS of exons 10 and 11, as predicted by RNA-Seq data. The protein encoded by *CTTN* gene, named cortactin, is localized in the cytoplasm and in areas of the cell-substratum contacts. Cortactin contains the following key domains: an amino-terminal acidic domain, a tandem repeat domain composed of six cortactin repeats, a carboxy-terminal proline-rich region that contains a number of phosphorylation sites and an SH3 domain. The new *CTTN* mRNA that lacks exons 10 and 11 encodes a predicted protein of 476 residues, whereas the annotated protein is of 550 amino acids (UniProt ID: Q14247). The lost amino acids (from 227 to 300) correspond to the fifth and sixth cortactin domains of the canonical protein (Figure S12).

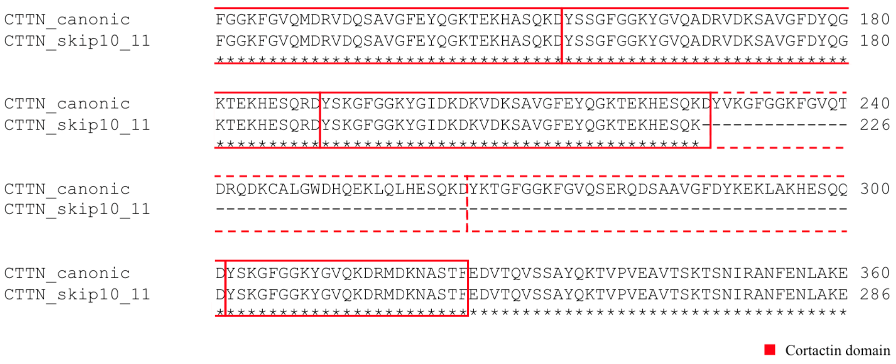


**Figure S12.** New CTTN protein alignment. A detail of the protein alignment of the canonical and the new cortactin protein isoform is shown. The dashed boxes represent the functional domains that are deleted in the novel protein sequence.

## Semaphorins and Plexins genes

Concerning *SEMA3C* gene, the RT-PCR showed, other than a 271 bp amplicon corresponding to the RefSeq canonic transcript (NM_006379), a product of 113 bp, whose length matches to the new AS transcript for *SEMA3C*. Direct Sanger sequencing confirmed for this isoform the skipping of exon 15. The new transcript overlaps the EST BF216749 and is supported by the AceView prediction SEMA3C.dAug10. The translation of this new transcript would lead to a protein composed of 514 aa, 237 aa shorter than the annotated one (UniProt ID: Q99985). In details, the protein alignment reveals that the two SEMA3C isoforms share amminoacids 1 to 495, whereas the C-terminal residues 496–514, translated in frameshift, totally differ compared to the canonic sequence (Figure S13). Similarly, the new *SEMA3F* transcript, lacking the exon 16, was detected by the presence of a 149 bp PCR product, shorter than the RefSeq annotated transcript (NM_004186.3), which is 307 bp long, and was confirmed by Sanger sequencing. *SEMA3F* new transcript is predicted to encode a protein of 571 aa, while the annotated protein (UniProt ID: Q13275) is 785 aa long (Figure 5D). The two SEMA3F isoforms share residues from 1 to 529. Class three semaphorins, among which Semaphorin3C and Semaphorin3F, are secreted molecules characterized by a large Sema domain, necessary for the homodimerization; by a PSI domain, which is common to Plexins, Semaphorins and Integrins; by an Immunoglobulin-like domain and a basic domain (rich in arginine and lysine), involved in the interaction with neuropilins, the obligate co-receptors of class 3 semaphorins. Both Semaphorin3C and Semaphorin3F novel isoforms lack the Ig-like C2 type domain and the R/K rich domain.

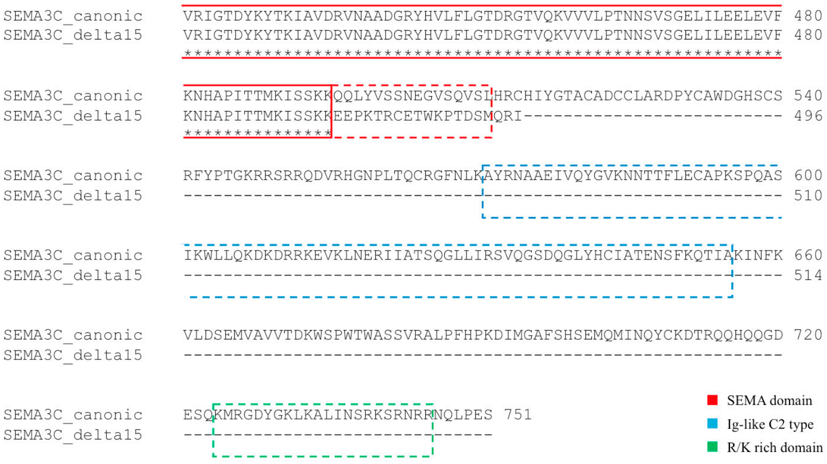


**Figure S13.** New SEMA3C protein alignment. A detail of the protein alignment of the canonical and the new semaphorin 3C protein isoform is shown. The dashed boxes represent the functional domains that are deleted in the novel protein sequence.

Finally, also for *PLXNB1* gene RT-PCR showed the presence of two amplicons, 332 bp and 159 bp long, respectively. The direct sequencing of the shorter PCR product revealed the skipping of exon 32 of the annotated transcript (NM_002673.5). PlexinB1 is the receptor for Semaphorin4D. It is known to be involved in axon guidance, invasive growth and cell migration through RhoA activation, with subsequent changes of the actin cytoskeleton [22]. The isoform 1 annotated in Uniprot is a single-pass type I membrane protein, while isoforms 2 and 3 are secreted proteins. The *PLXNB1* transcript that lacks the exon 32 is predicted to encode a protein composed of 1876 aa, sharing with the canonic protein (UniProt ID: O43157, 2135 aa long) the first 1859 residues. The remaining 276 amino acids are part of the cytoplasmic tail of PlexinB1, but Uniprot database doesn't report any annotated domain corresponding to that region (Figure S14).

```
PLXNB1_canonic      RTLDVEWRSGVAGHLILSDEDVTSEVQGLWRRLNTLQHYKVPDGATVALVPCLTKHVLRE 1860
PLXNB1_delta32      RTLDVEWRSGVAGHLILSDEDVTSEVQGLWRRLNTLQHYKVPDGATVALVPCLTKHVLRE 1860
                    ************************************************************

PLXNB1_canonic      NQDYVPGERTPMLEDVDEGGIRPWHLVKPSDEPEPPRPRRGSLRGGERERAKAIPEIYLT 1920
PLXNB1_delta32      NQDYVPGERHPAEVRG-------------------------------------------- 1876
                    *********

PLXNB1_canonic      RLLSMKGTLQKFVDDLFQVILSTSRPVPLAVKYFFDLLDEQAQQHGISDQDTIHIWKTNS 1980
PLXNB1_delta32      ------------------------------------------------------------

PLXNB1_canonic      LPLRFWINIIKNPQFVFDVQTSDNMDAVLLVIAQTFMDACTLADHKLGRDSPINKLLYAR 2040
PLXNB1_delta32      ------------------------------------------------------------

PLXNB1_canonic      DIPRYKRMVERYYADIRQTVPASDQEMNSVLAELSWNYSGDLGARVALHELYKYINKYYD 2100
PLXNB1_delta32      ------------------------------------------------------------

PLXNB1_canonic      QIITALEEDGTAQKMQLGYRLQQIAAAVENKVTDL 2135
PLXNB1_delta32      -----------------------------------
```

■ Lost amino acids

**Figure S14.** New PLXNB1 protein alignment. A detail of the protein alignment of the canonical and the new plexin-B1 protein isoform is shown. The dashed boxes represent the amino acids that are deleted in the novel protein sequence.

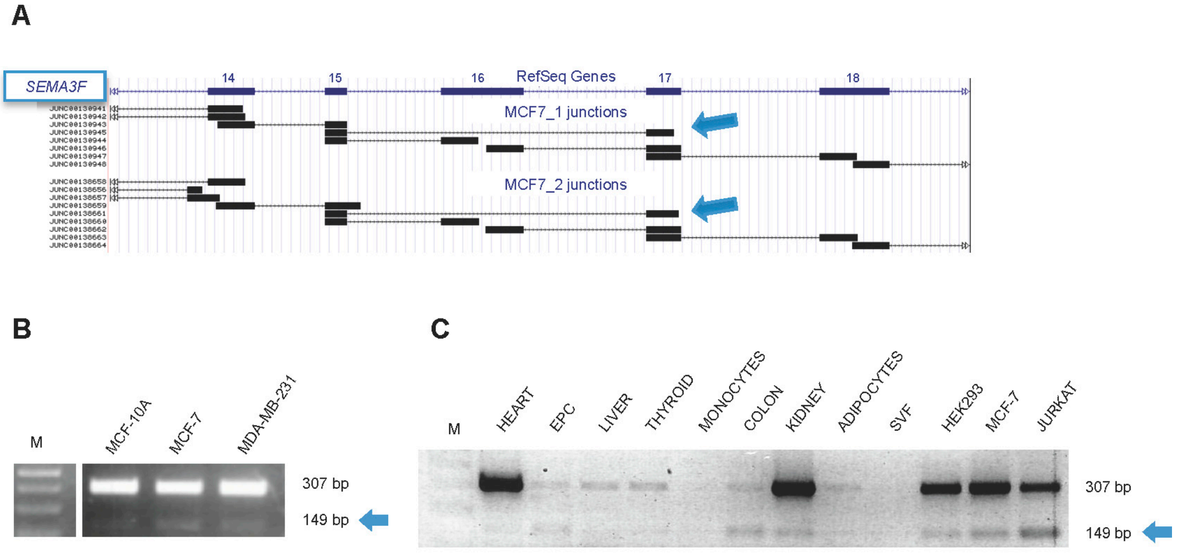


**Figure S15**. SEMA3F locus and mRNA expression. (**A**) Screenshot of UCSC Genome Browser Session containing expression track of our RNA-Seq experiment. Blue boxes indicate the annotated (RefSeq) exons, black boxes indicate the exon-exon junctions from the RNA-Seq experiment in both samples, the blue arrows indicate the junctions skipping exon 16; (**B**,**C**) Expression analysis of *SEMA3F* transcripts by RT-PCR on breast cell lines MCF-10A, MCF-7 and MDA-MB-231 and on a panel of human tissues and cell lines. The blue arrow indicate the new *SEMA3F* transcript missing exon 16. M indicates the 100bp DNA marker; EPC: endothelial progenitor cells; SVF: stromal vascular fraction; HEK293: human embryonic kidney cell line; JURKAT: human T lymphocyte cell line.
